# Quadruple Fenestrated Stentgrafts for Complex Aortic Aneurysms: Outcomes of Non-Stented Celiac Artery Fenestrations

**DOI:** 10.3390/jcm14155189

**Published:** 2025-07-22

**Authors:** Daniela Toro, Kim Bredahl, Katarina Björses, Tomas Ohrlander, Katja Vogt, Timothy Resch

**Affiliations:** 1 Department of Vascular Surgery, Rigshospitalet, Blegdamsvej 9, 2100 Copenhagen, Denmark; toro.dani89@gmail.com (D.T.); kim.kargaard.bredahl@regionh.dk (K.B.); katarina.anna-maria.bjoerses@regionh.dk (K.B.); katja.vogt@regionh.dk (K.V.); 2 Faculty of Health and Medical Sciences, University of Copenhagen, 2100 Copenhagen, Denmark

**Keywords:** complex abdominal aortic aneurysms (cAAA), quadruple fenestrated stentgrafts, non-stented celiac artery fenestrations, anatomical features of celiac artery, CA stenting

## Abstract

**Background**: Fenestrated stentgrafting has become a first-line treatment for juxtarenal aneurysms, and the incorporation of all renovisceral vessels with fenestrations has become common to increase the proximal sealing zone. This increases the complexity of the repair compared to using fewer fenestrations, and stenting of the celiac artery (CA), in particular, can be technically challenging. **Objective**: This study evaluates the mid-term outcomes of leaving the celiac artery unstented during quadruple fenestrated stentgrafting for complex aortic aneurysms. Additionally, it explores the clinical and anatomical factors that influence the decision to not stent the celiac artery. **Methods**: A retrospective review was conducted of patients with complex aortic aneurysms who underwent elective fenestrated endovascular aneurysm repair (FEVAR) between 2018 and 2023. Custom Cook Zenith grafts were used, and all patients underwent preoperative computed tomography angiography (CTA) as well as follow-up CTA to assess the celiac artery. This study evaluated celiac artery anatomic factors, such as proximal and distal diameter; presence of stenosis (<50% or >50%) and patency; length of any CA stenosis; CA takeoff angulation, CA tortuosity, early CA division; calcification; and presence of CA aneurysm or ectasia anatomical abnormalities. Recorded outcomes of CA instability included any stent stenosis, target vessel occlusion, reintervention, or endoleak (types 1C and 3). **Results**: A total of 101 patients underwent FEVAR, with 72 receiving a stent in the celiac artery and 29 not receiving it. Rates of technical success (96.5% vs. 100%), intervention times (256 min vs. 237 min), and lengths of hospital stay (5.1 vs. 4.7 days) were similar between unstented vs. stented groups. At one year, no significant difference in celiac artery instability was noted (17.2 vs. 5.5%; *p* = 0.06). Risk factors for CA occlusion on univariate analysis included a steep takeoff angle (≥140°), length of stenosis >6.5 mm, proximal diameter ≤6.5 mm, preoperative stenosis ≥50%, and celiac artery tortuosity. **Conclusions**: Anatomical features of the CA impact the ability to achieve routine CA stenting during FEVAR. Selectively not stenting the celiac artery during FEVAR might simplify the procedure without compromising patient safety and mid-term outcomes.

## 1. Introduction

Complex abdominal aortic aneurysms (cAAA) require branched or fenestrated endografts for endovascular repair [1,2]. Technical advances over the past two decades have improved the procedure but target vessel (TV) stenting can still pose a significant challenge [3,4,5,6]. TV tortuosity, stenosis, small diameter, highly angulated sharp takeoff and early branching increase catheterization difficulty and the risk of intraoperative complications and technical failures [7,8].

Furthermore, failure of TV catheterization is associated with increased mortality and morbidity [8], and stenting of small-diameter TVs increases the risk of in-stent restenosis. Damaged stents and in-stent restenosis contribute to late TV occlusion, which is observed in up to 10% of the procedures performed after two years [9].

The celiac artery (CA) can be particularly challenging to catheterize during fenestrated EVAR (FEVAR) due to its specific anatomical characteristics such as stenosis, angulation, and early division [10]. In addition, the celiac artery is affected by respiratory movements and the diaphragmatic crus, which can compromise the integrity of the stent and increase the risk of TVI and postoperative occlusion [11,12].

Leaving a CA fenestration unstented has recently been proposed to simplify multivessel FEVAR, thus reducing operative time and radiation exposure [10]. Follow-up indicates that this approach might not result in significant differences in reintervention rate, reintervention-free survival, endoleak, or mortality compared to stenting the CA [13].

The purpose of the current study is to evaluate the outcomes of not stenting the celiac artery during FEVAR in juxtarenal, pararenal, and type 4 TAAA and its effect on mid-term TV instability, patency, and reintervention. Furthermore, we aim to investigate the reasons behind failure to stent the CA from a clinical and anatomical standpoint.

## 2. Materials and Methods

This is a single-center, retrospective study of patients with juxtarenal, pararenal, and type 4 TAAA who underwent elective FEVAR incorporating four visceral target vessels between April 2018 and October 2023. All FEVAR grafts used were custom-made devices based on the Cook Zenith platform (COOK Medical, Bjævreskov, Denmark), using either preloaded or standard delivery systems. Device design and implantation technique have been extensively described previously [14,15].

Preoperative demographics, anatomic aneurysmal characteristics, procedural details, and perioperative complications were collected.

Clinical data was extracted from electronic medical records. All patients underwent preoperative computed tomography angiography (CTA) as well as follow-up CTA and Doppler ultrasound imaging at 3 and 12 months postoperatively. Additional imaging studies, such as interval visceral doppler ultrasound and contrast-enhanced ultrasound (CEUS), were performed based on clinical indications.

Arterial phase CTAs were conducted following a standardized protocol and were reconstructed with 0.5 to 2 mm axial slices. Image post processing was performed using a dedicated 3D workstation (Aquarius, TeraRecon, Durham, NC). Vessel diameters were measured on orthogonal reconstructions perpendicular to a centerline along the axis of blood flow.

**Figure 1:**
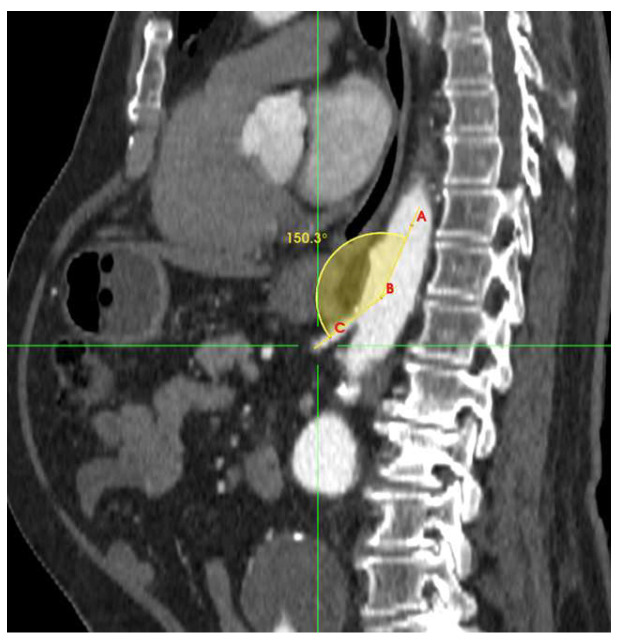
CA tortuosity (defined as the tortuosity index calculated as the ratio of the centerline length from the celiac artery to the division of the celiac artery divided by a straight line between two end points); early CA division; calcification; and presence of CA aneurysm or ectasia. Other anatomical abnormalities were also noted (A-B-C denotes the seed points for angulation determination).

Multiple anatomical parameters of the celiac artery were evaluated on preoperative CTAs according to reporting standards [16], including proximal and distal diameter; presence of stenosis (<50% or >50%) and patency; length of any CA stenosis; and CA takeoff angulation (defined as the angle between the longitudinal axis of the abdominal aorta and the longitudinal axis of the celiac artery, Figure 1).

Clinical and imaging follow-up assessed CA patency, endoleaks, and reinterventions in the celiac artery.

### 2.1. Outcomes

The cohort was divided into two groups for analysis: those with and without stent placement in the CA during 4 vessel FEVAR. Branch vessel instability was defined by a composite of any stent stenosis, target vessel occlusion, reintervention, or endoleak (types 1C and 3) as described by Mastracci et al. [17]. CA reintervention-free survival, CA endoleak-free survival, and mortality rates were also compared between groups.

### 2.2. Statistical Analysis

Baseline and operative characteristics, perioperative mortality, and postoperative complications were compared using univariate analysis stratified by unstented and stented celiac artery (CA) during FEVAR.

Continuous variables were evaluated for normal distribution via visual inspection of scattergrams and presented as mean ± standard deviation. For continuous variables, comparisons between groups were made with two-sided *t*-tests or Wilcoxon rank-sum tests. For categorical variables, χ^2^ and Fisher tests were used, depending on the sample size.

Event rates were calculated based on Kaplan–Meier censoring estimates used for CA reintervention/endoleak-free survival and to estimate survival probability and are presented as cumulative incidences. The log-rank test was used to compare survival curves between groups.

Statistical analysis was performed using SPSS software (IBM SPSS Statistics for Windows, Version 27, IBM Corp., Armonk, NY, USA), and significance for all tests was assumed at *p* value < 0.05.

## 3. Results

A total of 101 patients underwent four-vessel fenestrated endovascular aneurysm repair (FEVAR) with or without stent placement in the celiac artery (CA) during the study period. Of these, 72 patients received a CA stent, while 29 did not. The demographic characteristics between the stented and unstented groups were comparable (Table 1).

Anatomy: Significant anatomical differences in the celiac artery were observed preoperatively between the two groups (Table 2).

The unstented group exhibited more complex anatomical features, including a highly angulated CA takeoff (≥140°) in 68.9% of cases, a proximal CA diameter of ≤6.5 mm in 34.4%, CA stenosis of ≥50% in 62.0%, and a stenosis length > 6.5 mm in 68.9%. Additionally, CA tortuosity was noted in 55.1% of the unstented group.

Procedural Details: Preoperative aortic diameter was similar between the groups. All procedures were performed under general anesthesia. FEVAR devices were implanted via percutaneous transfemoral access, using either preloaded (37.6%) or standard (62.3%) delivery systems. Fenestrations and target vessels not accessed with a preloaded catheter were routinely accessed using a steerable sheath (Tourguide 7F, Medtronic, Santa Rosa, USA) via a 16–18 F contralateral sheath placed into the main FEVAR device (Table 3).

The decision to not stent the CA was made intraoperatively in all cases based on a combination of failed stenting and the following prerequisites: (1) preoperative imaging had determined an adequate sealing zone caudal to the SMA, (2) wall apposition of the celiac fenestration was present, and (3) no endoleak was seen in the absence of a bridging stent placed into the celiac artery. In eight cases (27.6%), this decision was influenced by significant challenges in catheterizing the CA. In another 20 cases (69.0%), difficulties in CA catheterization led to kinking or breaking of the steerable sheath, resulting in failed stenting. In one case (3.4%), the CA was intentionally left unstented due to adequate sealing and fenestration apposition (Table 3).

Stent Types: In the stented group, various stent types were used, including BeGraft (Bentley Innomed) in 59.7% of cases, Advanta (Getinge) in 20.8%, VBX (Gore) in 18.0%, and BeGraft Plus (Bentley Innomed) in 1.4% (Table 3).

Technical Success and Postoperative Outcomes: There was no significant difference in primary technical success, intervention time, or fluoroscopy time between the stented and unstented groups (Table 3). The length of hospital stay was similar.

In the stented group, three early reinterventions were required for bleeding: two in the CA and one in the superior mesenteric artery (SMA). These were guidewire-related injuries and were successfully treated with selective coil embolization.

In the unstented group, there was one case of grade 1 spinal cord ischemia (3.4%) in a patient with a pararenal aneurysm, which was successfully treated with spinal drainage.

In the stented group, there was one case of grade 0 spinal cord ischemia in a patient with an IV thoracoabdominal aneurysm and another case of grade 0 in a patient with a juxtarenal aneurysm; neither required postoperative spinal drainage. The occurrence of adverse events did not significantly differ between the groups (Table 4).

Renal injury, as classified by the Rifle score, occurred in 10.3% of patients in the unstented group compared to 5.6% in the stented group (*p* = 0.40) [18]. Three cases (4.2%) of bowel ischemia were observed in the stented group, caused by SMA stent occlusion secondary to stent compression. All were successfully treated with emergent SMA stent relining, with only one patient requiring bowel resection (Table 4).

Three-Month Follow-Up: At 3 months, celiac artery instability was 13.7% in the unstented group and 2.7% in the stented group. Among patients with preoperative CA stenosis of less than 50%, 10.3% progressed to stenosis greater than 50%. In the stented group, the proportion of arteries without significant stenosis increased from 36% preoperatively to 93% post-stenting, with no residual stenosis after stent placement. One case of stent occlusion, one case of graft stenosis, and two deaths were recorded at this follow-up. No patients with TVI in the unstented group had any clinical symptoms or signs of CA-related ischemia.

Branch Instability and Endoleaks: At one year, no statistically significant difference in CA instability was observed between the groups. The unstented group exhibited a 17.2% rate of CA instability due to an increase in occlusions (n = 4). In the stented group, the instability rate was 5.5%, with two cases of stent occlusion and two cases of in-stent stenosis (Table 5).

All celiac artery occlusions were asymptomatic, with no signs of mesenteric ischemia or liver function abnormalities. No significant difference in non-CA vessel instability was noted between the groups, with all cases of instability related to the renal artery (Table 6).

**Figure 2:**
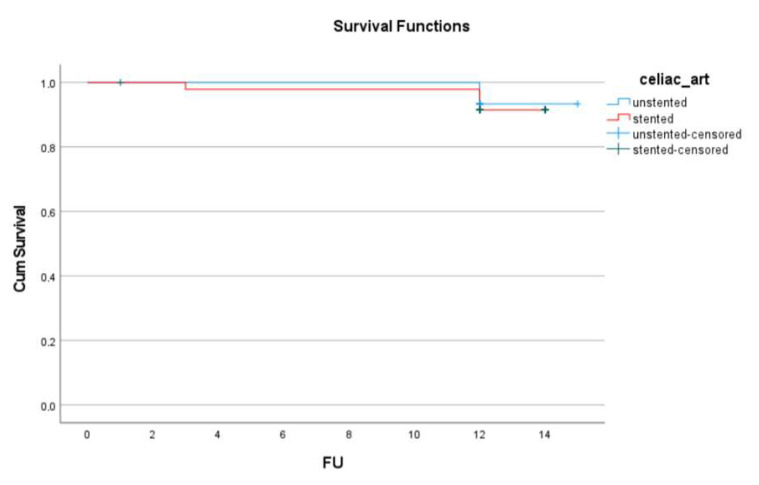
Reintervention free survival at follow up.

Mortality and Reintervention-Free Survival: Six deaths occurred during the follow-up period (one in the unstented group and five in the stented group), with no significant difference between the groups (Figure 2).

**Figure 3:**
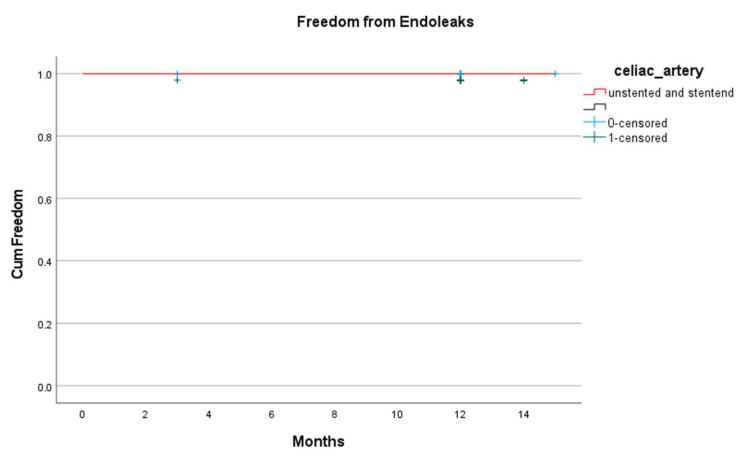
Endoleak free survival during follow up.

**Figure 4:**
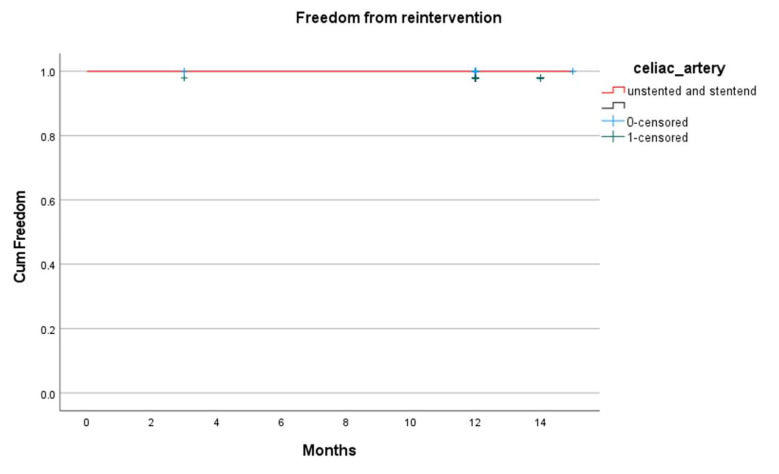
Celiac artery reintervention during Follow up.

The celiac artery reintervention-free survival was 100%, with no recorded cases at 3 months or 1 year (Figure 3). No celiac artery-related endoleaks were observed at either follow-up (Figure 4).

Risk Factors for CA Occlusion: Due to the low number of events in the unstented group, no risk factor prediction could be performed. The descriptive findings presented in Table 2 suggest that the CAs that were not stented had more adverse features but the actual impact of this on the CA outcome could not be further elucidated.

## 4. Discussion

This study demonstrated that in patients with complex aortic aneurysms, leaving the celiac artery fenestration unstented during FEVAR did not lead to a significant increase in celiac artery instability at one-year follow-up. Furthermore, an unstented CA fenestration did not lead to significant increases in mortality, endoleaks, or CA reinterventions.

Over the past decade, the adoption of four-vessel FEVAR designs has reduced type Ia endoleaks to <1% [19], likely due to the extended proximal sealing zone. Incorporating the celiac artery with a fenestration rather than a scallop also allows future proximal extension of the endograft if needed. Studies have shown that this strategy of increasing aortic coverage does not increase rates of hospital complications or mortality, nor does it increase radiation exposure or reduce technical success [13,20,21]. Witheford et al. [13] argue that increasing aortic coverage reduces the long-term risk of non-CA vessel instability compared to shorter devices.

Incorporating the CA with an unstented fenestration does, however, seem to increase CA instability at follow-up. It does not, however, result in significant differences in terms of reintervention rate, reintervention-free survival, incidence of endoleak, or mortality compared to using a stented CA fenestration.

In contrast, our data did not show a significant increase in CA instability compared to the stented group at one-year follow-up (17.2% vs. 5.5%). Even though the unstented group had an increase in cases of clinically silent occlusions, this did not achieve statistical significance (*p* = 0.054). Due to the low number of patients and events, this might be a type 2 error; thus, a larger data set might reveal a significant difference. Like other studies, the overall target vessel (TV) instability primarily involved renal artery fenestrations. Renal target vessels are more prone to complications than visceral arteries in F/BEVAR, with a complication rate of 6% versus 2% [22]. No CA-related endoleaks or reinterventions were observed in either group, and reinterventions on the CA after FEVAR and BEVAR remain rare [13,23]. A recent study indicated that non-stenting of the CA had a low reintervention rate, despite a higher frequency of occlusion, most of which remain clinically silent [10].

In the current study, the decision not to stent the CA was made intraoperatively, often after extensive attempts had been made to catheterize the CA and place a stent. Before abandoning celiac artery stenting, each case was evaluated regarding the ability to achieve an adequate aneurysm seal in the absence of completing the celiac artery stenting. This included assessing the preoperative sealing zone below the SMA as well as establishing that there was wall apposition of the unstented celiac fenestration to the celiac artery orifice. In addition, an angiogram ruling out an endoleak from the unstented celiac fenestration was performed before stenting attempts were abandoned. If these criteria are not met, further attempts at celiac stenting should be performed. Unfortunately, the retrospective nature of this study did not allow for more granular information regarding other reasons for abandoning CA stenting, whether multiple stents were attempted or whether a particular stent was more prone to fail in the CA.

Notably, there was no significant difference in operative or fluoroscopy time between the groups despite the seemingly more complex anatomy in the unstented group. This finding might suggest that in the unstented group, complex anatomical features and unsuccessful attempts at stent placement contributed to prolonged procedure duration despite no stent being placed, as the actual stenting step, once a stable position is reached, is not long. Cases of perioperative bowel ischemia secondary to SMA stent obstruction and bleeding from the CA and SMA were confined to the stented group, underscoring the risks associated with excessive manipulation in the context of unstable guidewire positions and visceral stents protruding into the main body of a stentgraft. Although cone-beam CT was routinely used to evaluate stent patency, it seems apparent that this imaging modality may not entirely detect potential issues with target vessel stents due to its lower resolution compared to standard CTA. Regarding TV bleeding, this is often overlooked due to vasospasm. TVs that are difficult to access or that provide limited guidewire stability due to complex anatomy force riskier wire positioning to enable stent placement, which clearly carries a risk.

Preoperative anatomy plays a crucial role in the ability to successfully stent a CA. Our analysis identified significant anatomical differences between the stented and unstented groups, with the latter displaying more complex characteristics, such as a more angulated CA takeoff, smaller proximal diameter, and a higher prevalence of significant stenosis. These factors not only make catheterization more challenging but also limit the ability to place sheaths and stents into the CA. Furthermore, smaller vessel diameters may restrict the selection of appropriate stents, potentially increasing the risk of occlusion or restenosis. In this study, these anatomical challenges were associated with catheterization failure (27.6%) and difficulties in sheath and stent placement (69%) in the CA, which in turn correlated with longer procedure durations. In the literature, cannulation/stenting failure and prolonged procedures are associated with increased mortality and morbidity. Therefore, it seems important that target vessel cannulation and stenting be performed expeditiously [8].

Furthermore, we also found an association between anatomical risk factors and CA occlusion in both the stented and unstented groups. The effects of preoperative CA stenosis > 50% on TV outcome after F/BEVAR have been associated with reduced primary patency and lower freedom from target vessel instability in previous studies [24]. Our analysis highlighted that a proximal CA diameter ≤ 6.5 mm is an independent predictor of both occlusion and the development of significant stenosis (≥50%) during FU. Stenting small target vessels increases the risk of in-stent restenosis, which has been associated with late TV occlusion in up to 10% of target vessels after two years [9]. These findings suggest that in select cases, avoiding stent placement in the CA during FEVAR might be beneficial, especially when considering anatomical complexities. This approach could simplify the procedure, effectively converting a 4FEVAR into a 3FEVAR, while reducing radiation exposure, contrast volume, and procedural costs. Additionally, this strategy may facilitate future repairs involving proximal disease extension, as it allows for the implantation of thoracic stentgrafts that seal in the previous fenestrated stentgraft, covering or incorporating the CA fenestration if needed [10]. Future studies with larger cohorts of prospective data, perhaps enhanced by AI analysis and deep learning algorithms, might provide a better understanding of the situations that mandate target vessel incorporation into a repair in relation to expected procedural failure and overall risk management in the short- and mid-term. It might also aid and improve intraoperative decision making.

### Limitations

This study has several limitations, including a small sample size and limited follow-up. This limits the conclusions to be drawn and introduces the possibility of a type 2 error with regards to the presence of occlusions in the unstented celiac artery. Although not statistically different from the stented group, the low numbers might account for this. In addition, outcomes after 1 year are not known, and it is possible that target vessel instability might increase with a longer follow-up. The retrospective design is inherently prone to selection bias. Additionally, multiple operators with varying levels of experience were involved, which may have influenced the results. The small sample size also precluded a detailed analysis of the impact of specific target vessel stents and accessories on TV outcomes.

## 5. Conclusions

The anatomical features of the CA seem to impact the feasibility of routine stenting during FEVAR. Selectively not stenting the CA may simplify the procedure and may not compromise patient safety or mid-term outcomes. Further studies with larger patient cohorts and longer follow-up periods are necessary to validate the safety and efficacy of this approach.

## Figures and Tables

**Table 1:** Baseline demographics and preoperative morphology of the 101 patients.

Variables	Unstented (n = 29)	Stented (n = 72)	*p* Value
Age	74.1 ± 5.3	73.8 ± 5.6	0.83
Male sex	26 (89.7)	63 (87.5)	0.75
Hypertension	19 (65.5)	53 (73.6)	0.41
Diabetes	2 (6.9)	10 (13.9)	0.32
Chronic kidney disease	7 (24.1)	23 (31.94)	0.43
Coronary artery disease	9 (31.0)	26 (36.1)	0.62
Peripheral arterial occlusive disease	5 (17.2)	10 (13.9)	0.67
Smoking status			0.0001
Never	2 (6.9)	6 (8.3)	
Former	13 (44.8)	45 (62.5)	
Current	14 (48.3)	21 (29.2)	

**Table 2:** Preoperative anatomical features of the celiac artery.

Preoperative Anatomical Features of CA Univariate Analysis	Unstented (n = 29)	Stented (n = 72)	*p* Value
Takeoff angulation			0.0184
<140°	9 (31.0)	41 (56.9)	0.018
≥140°	20 (68.9)	31 (43.0)	0.019
Proximal diameter			0.0005
≤6.5 mm	10 (34.4)	5 (6.9)	0.0001
>6.5 mm	19 (65.5)	67 (93.0)	0.025
Distal diameter			0.388
≤6.5 mm	9 (31.0)	29 (40.2)	0.285
>6.5 mm	20 (68.9)	43 (59.7)	0.424
Patency overall			0.0029
No Stenosis	3 (10.3)	26 (36.1)	0.009
Stenosis < 50%	8 (27.5)	26 (36.1)	0.414
Stenosis ≥ 50%	18 (62.0)	20 (27.7)	0.001
Length of stenosis			0.00001
≤6.5 mm	9 (31.0)	38 (52.7)	0.02
>6.5 mm	20 (68.9)	34 (47.2)	0.05
Calcification	19 (65.5)	46 (63.8)	0.87
Early division (main trunk < 6 mm)	1 (3.4)	0	0.11
Artery Tortuosity	16 (55.1)	14 (19.4)	0.00037
Aneurysm	0	1 (1.3)	0.52
Anatomical Abnormalities	4 (13.7)	6 (8.3)	0.40

**Table 3:** Preoperative and perioperative characteristics.

Preoperative and Perioperative Characteristics	Unstented (n = 29)	Stented (n = 72)	*p* Value
cAAA type			
Type IV	1 (3.4)	1 (1.4)	0.51
Paravisceral	2 (6.9)	0	0.025
Pararenal	10 (34.5)	11 (15.3)	0.032
Juxtarenal	16 (55.1)	60 (83.3)	0.003
Preoperative aortic diameter -mm	68.9 ± 12.7	67.3 ± 9.8	0.49
Cook zenith fenestrated CMD			
standard	15 (51.7)	48 (66.7)	0.16
preloaded	14 (48.3)	24 (33.3)	0.16
General anesthesia	29 (100.0)	72 (100.0)	ns
Access site			
percutaneous femoral access	29 (100)	72 (100)	ns
percutaneous brachial access	4 (13.7)	3 (4.2)	0.91
percutaneous axillary access	1 (3.5)	1 (1.4)	0.50
Intraoperative CA complication			
failed catheterization	8 (27.6)	0	0.0001
unsuccessful attempted stenting	20 (69.0)	0	<0.0001
Stenting in CA was deemed unnecessary	1 (3.4)	0	0.11
intraoperative endoleak from CA	1 (3.4)	0	0.11
CA bridging stent			
BeGraft		43 (59.7)	na
BeGraft plus		1 (1.39)	na
VBX		13 (18.0)	na
V12 Advanta		15 (20.8)	na
Technical success for CA	28 (96.5)	72 (100.0)	0.11
Primary Technical success	28 (96.5)	65 (90.2)	0.29
Total operating time min	256 ± 93.8	237.1 ± 79.7	0.30
Fluoroscopy time-min	104.4 ± 40.5	103.4 ± 39.5	0.90

**Table 4:** Postoperative characteristics.

Postoperative Characteristics	Unstented (n = 29)	Stented (n = 72)	*p* Value
In-hospital (days)	5.1 ± 5.5	4.7 ± 4.7	0.71
Early reintervention for endoleak or migration—before discharge	0	0	na
Early reintervention for rupture or bleeding—before discharge	1 (3.4)	4 (5.6)	0.64
Reintervention (central arterial perforation)	0	3 (4.2)	0.26
Hematoma	0	1 (1.4)	0.52
False aneurysm	1 (3.4)	0	0.11
Early reintervention for lower limb ischemia Access-site, device, and procedure-related.	1 (3.4)	3 (4.2)	0.85
Postoperative adverse events			
spinal cord ischemia	1 (3.4)	2 (2.8)	0.87
mesenteric ischemia	0	3 (4.2)	0.26
renal ischemia/failure	3 (10.3)	4 (5.6)	0.40
aortic dissection	0	1 (1.4)	0.52
cerebral event	0	1 (1.4)	0.52
cardiac event	0	2 (2.8)	0.36
pulmonary event	0	1 (1.4)	0.52
multiorgan failure	0	1 (1.4)	0.52
intensive care > 1 day	0	1 (1.4)	0.52
other	2 (6.9)	1 (1.4)	0.14
Early reintervention for access-site lymphocele and/or infection	0	0	na

**Table 5:** Celiac artery follow-up at 1 year.

	Unstented (n = 29)	Stented (n = 72)	*p* Value
<50% Stenosis	1 (3.4)	0	0.11
≥50% Stenosis	0	0	na
CA Occlusion	4 (13.7)	2 (2.7)	0.034
CA stent stenosis	NA	2 (2.7)	na
Symptoms	0	0	na
Lost to Fu	4 (13.7)	19 (26.3)	0.17
No info	1 (3.4)	0	0.11
Endoleaks	0	0	na
Reinterventions	0	0	na
Death	1 (3.4)	5 (6.9)	0.50
CA instability	5 (17.2)	4 (5.5)	0.06

**Table 6:** Non-CA target vessel follow-up at 12 months.

	Unstented (n = 29)	Stented (n =72)	*p* Value
Endoleaks			
Type Ic	1 (3.4)	0	0.11
Type III	0	0	na
Branch stenosis	0	0	na
Branch occlusion	1 (3.4)	2 (2.7)	0.85
Branch reintervention	1 (3.4)	0	0.11
Non CA TV instability	3 (10.3)	2 (2.8)	0.11

## Data Availability

The original contributions presented in this study are included in the article. Further inquiries can be directed to the corresponding author(s).
